# Supplementary material for: Content-rich biological network constructed by mining PubMed abstracts
Source: BMC Bioinformatics. 2004 Oct 8;5:147. doi: 10.1186/1471-2105-5-147 (PMC528731; doi:10.1186/1471-2105-5-147)
Supplement: Additional File 5 — The original Chilibot query results of the term "long-term potentiation (LTP)" and 22 other terms, limiting the latest references analyzed to the years 1990, 1995, 2000, and 2004. [file 1471-2105-5-147-S5.bz2 › chilibotAdditionalFile5/ltp1995/html/ARC_ACTININ.html]

 


 **ARC** and **ACTININ** 
  
Found 2 abstracts in PubMed,  **2 abstracts were retrieved and analyzed**.  


---

 Search Google  |
 PDF files only 
|  EDU domain only 

---

**Interactive relationship** (e.g. stimulation, inhibition, etc)

- Interference reflection microscopy demonstrated that the spread cells were attached to the substratum in  **arc**  shaped regions, which corresponded to arcs containing alpha  **actinin**  as seen by specific immunofluorescence of the same cells.  Ref: 6373330 Exp Cell Res, 1984
- In addition to actin, the  **ARC**  was enriched with proteins that showed cross reactivity to antibodies to alpha  **actinin**  and the 50K actin binding protein elongation factor 1 alpha from Dictyostelium.  Ref: 1340274 Arch Med Res, 1992
